# P-47 - SOCIAL AND DEMOGRAPHIC PATTERNS IN INFLUENZA VACCINE UPTAKE AMONG 18–64 YEAR-OLDS WITH CLINICAL RISK CONDITIONS IN SCOTLAND: DESCRIPTIVE FINDINGS FROM THE 2025/26 SEASON

**DOI:** 10.1093/pubmed/fdag046.085

**Published:** 2026-07-09

**Authors:** Dr Allan James, Dr Leonie Hunter, Mr Graham McGowan, Dr Kirsty Morrison, Dr Christopher Sullivan, Dr Claire Cameron, Professor Sam Ghebrehewet, Dr Cheryl Gibbons

**Affiliations:** Public Health Scotland; Public Health Scotland; Public Health Scotland; Public Health Scotland; Public Health Scotland; Public Health Scotland; Public Health Scotland; Public Health Scotland

## Abstract

**Background:**

Seasonal influenza vaccination is recommended for adults with clinical risk conditions due to increased risk of severe outcomes. In Scotland, uptake among adults aged 18–64 years is routinely reported, but not differentiated by clinical risk group. Descriptive analyses are needed to understand variation in uptake and to identify populations for improvement activity. Seasonal influenza vaccination is recommended for adults with clinical risk conditions due to increased risk of severe outcomes. In Scotland, uptake among adults aged 18–64 years is routinely reported, but not differentiated by clinical risk group. Descriptive analyses are needed to understand variation in uptake and to identify populations for improvement activity.

**Objectives:**

To describe crude influenza vaccine uptake during the 2025/26 season among adults aged 18–64 years with clinical risk conditions, examining variation by clinical risk group, age, sex, and socioeconomic deprivation.

**Methods:**

Using Scottish primary care data, adults aged 18–64 years eligible for influenza vaccination in 2025/26 with at least one recorded clinical risk condition were identified. Eligibility was grouped into nine categories aligned with the Joint Committee on Vaccination and Immunisation (JCVI) defined groups. Crude uptake was calculated as the proportion vaccinated within each category and described by age, sex, Scottish Index of Multiple Deprivation (SIMD) quintile, and number of recorded conditions.

**Results:**

Across clinical risk groups, crude uptake ranged from approximately one third to one half of eligible individuals. Uptake was lowest among younger adults and increased with age. Females had higher uptake than males, with the largest sex differences in younger age bands. A marked socioeconomic gradient was evident: uptake was lowest in the most deprived areas and highest in the least deprived. Uptake also increased with the number of recorded conditions across all deprivation levels.

**Conclusions:**

Overall vaccine uptake for the at-risk category was 38.9% for the winter 2025/26 influenza vaccination programme. Results from this study provide uptake of those with clinical risk conditions that contribute to the overall statistic, highlighting sub-populations with the lowest uptake and where potential improvements could be made.

